# Prevalence of Benign Prostatic Hyperplasia and Prostate Cancer Among Men Presenting with Lower Urinary Tract Symptoms at a Tertiary Referral Hospital in Dar es Salaam, Tanzania: A Retrospective Cross-Sectional Study

**DOI:** 10.3390/jcm15082914

**Published:** 2026-04-11

**Authors:** Alaa Imad Ali Amin, Lara M. Samhan, Abdul Rehman Zia Zaidi, Akram Imad Ali Amin, Zainudheen Faroog, Bedour Sulaiman Raddad Almalki, Baraa Alghalyini

**Affiliations:** 1 College of Medicine, University of Medical Sciences and Technology, Khartoum P.O. Box 12810, Sudan; 2 College of Medicine, Alfaisal University, Riyadh 11533, Saudi Arabia; 3 Department of Family & Community Medicine, College of Medicine, Alfaisal University, Riyadh 11533, Saudi Arabia; balghalyini@alfaisal.edu

**Keywords:** lower urinary tract symptoms, benign prostatic hyperplasia, prostate cancer, international prostate symptom score, prostate-specific antigen, Tanzania, sub-Saharan Africa

## Abstract

**Background:** Lower urinary tract symptoms (LUTSs) are among the most common urological complaints in older men, frequently arising from benign prostatic hyperplasia (BPH) or prostate cancer (PCa). While both conditions share overlapping symptomatology, the way each condition progresses and is managed differs considerably. In sub-Saharan Africa, data on the relative burden of BPH and PCa among men presenting with LUTSs are scarce. This study aimed to determine the prevalence of histologically confirmed BPH and PCa among men presenting with LUTSs at a major tertiary referral center in Tanzania and to explore the association between specific urinary symptoms and histopathological diagnoses. **Methods:** A retrospective cross-sectional study was conducted at Muhimbili National Hospital (MNH), Dar es Salaam, Tanzania, reviewing medical records of adult male patients aged ≥50 years who presented with LUTSs and underwent prostatic biopsy between January and December 2023. A total of 133 patients were included through simple random sampling from an eligible population of 260. Data on demographics, comorbidities, International Prostate Symptom Score (IPSS), serum prostate-specific antigen (PSA), prostate volume, and histopathological biopsy outcomes were extracted using a purpose-built digital form. This study was conducted in compliance with the Strengthening the Reporting of Observational Studies in Epidemiology (STROBE) guidelines. **Results:** Most patients (39.8%) were aged 70 to 79 years. Hypertension was the most frequent comorbidity among those with chronic disease (31.65%), followed by diabetes mellitus (12.03%). The mean serum PSA was 465.1 ng/mL (SD = 1610.1), and the mean prostate volume was 80.6 cm^3^ (SD = 75.6). Histopathologically, 57.9% of biopsies were benign and 40.6% were malignant. The most commonly reported IPSS symptoms were urinary frequency (78.2%), weak stream (78.2%), and incomplete emptying (64.7%). Most patients (59.4%) had severe IPSSs. Statistically significant associations were observed between biopsy outcomes and incomplete emptying (*p* = 0.011), frequency (*p* = 0.014), weak stream (*p* = 0.022), nocturia (*p* = 0.001), urge incontinence (*p* = 0.004), and post-void dribbling (*p* < 0.001). IPSS severity was significantly associated with biopsy diagnosis (*p* < 0.001), with 63% of malignant cases presenting with moderate symptom scores. **Conclusions:** BPH was the predominant histopathological diagnosis among men presenting with LUTSs at this tertiary center, while prostate cancer accounted for a substantial minority of cases. Certain individual LUTSs, particularly nocturia, urge incontinence, and post-void dribbling, demonstrated significant associations with malignant histopathology. These findings underscore the necessity for systematic histopathological evaluation in all men presenting with LUTSs in resource-limited settings, irrespective of symptom severity.

## 1. Introduction

Lower urinary tract symptoms (LUTSs) encompass a broad spectrum of storage, voiding, and post-micturition symptoms that can substantially affect quality of life in older men [1,2]. Clinically, these symptoms are broadly categorized into irritative (storage) manifestations, including urinary frequency, urgency, nocturia, and urge incontinence, and obstructive (voiding) symptoms such as hesitancy, weak urinary stream, straining, intermittency, and the sensation of incomplete bladder emptying [1]. Although LUTSs are not inherently life-threatening, over time, untreated LUTSs can cause meaningful functional decline and psychological burden [2].

The prostate gland, a walnut-sized exocrine organ encircling the prostatic urethra, sits in a position that makes it central to the development of LUTSs [3]. Benign prostatic hyperplasia (BPH), characterized by non-malignant stromal and glandular proliferation within the transition zone of the prostate, represents the most frequent etiology of LUTSs in older men [4,5]. Histological evidence of BPH becomes increasingly common with age, affecting approximately 50% of men by the sixth decade and exceeding 80% by the ninth decade of life [6]. Established risk factors for BPH include advanced age, family history, metabolic syndrome, type 2 diabetes mellitus, cardiovascular disease, and physical inactivity [5,7]. Although complications such as acute urinary retention and obstructive nephropathy are not common, both carry considerable morbidity; acute retention may necessitate emergency catheterization, while obstructive nephropathy can progress to irreversible renal impairment if bladder outlet obstruction remains unrelieved [5].

Prostate cancer (PCa), defined as a malignant neoplasm originating in the prostatic epithelium, is the second most frequently diagnosed cancer and the fifth leading cause of cancer-related mortality among men worldwide [8]. According to GLOBOCAN 2022 estimates, prostate cancer accounted for approximately 1.47 million new diagnoses and 397,000 deaths worldwide [8]. In sub-Saharan Africa (SSA), the burden of prostate cancer in this region is especially worrying; prostate cancer is the most commonly diagnosed malignancy among men in the region, and mortality is disproportionately high, driven mainly by late presentation and restricted access to diagnosis and treatment [9,10]. In Tanzania specifically, hospital-based studies have documented that a substantial proportion of prostate cancer patients present with locally advanced or metastatic disease [11,12].

Both BPH and PCa can manifest with overlapping LUTSs, a problem compounded in settings where advanced urological workup is not routinely available [13]. The International Prostate Symptom Score (IPSS), a validated eight-item self-administered questionnaire, provides a validated, standardized measure of symptom severity to guide clinical assessment [14]. However, the IPSS alone cannot reliably distinguish between benign and malignant prostatic pathology, necessitating adjunctive evaluation with serum prostate-specific antigen (PSA) measurement and histopathological confirmation through prostatic biopsy [15].

Despite the well-established global burden of BPH and PCa, epidemiological data from low- and middle-income countries (LMICs) in SSA remain comparatively sparse [16,17]. A recent scoping review confirmed that evidence on the burden, quality-of-life impact, and treatment patterns of BPH in SSA is still notably thin [16]. Furthermore, the rising absolute burden of BPH in LMICs, driven by population aging and increasing life expectancy, makes locally grounded data all the more necessary for planning services and allocating resources effectively [18,19]. Within Tanzania, Muhimbili National Hospital (MNH) in Dar es Salaam serves as the country’s largest tertiary referral, research, and teaching hospital, functioning as the primary center for the management of complex urological conditions [20,21].

Against this background, the present study aimed to determine the prevalence of histologically confirmed BPH and PCa among men presenting with LUTSs at MNH and to explore potential associations between specific LUTSs, symptom severity, comorbid conditions, and histopathological biopsy outcomes. We hope these findings will help fill a real gap in our understanding of prostatic disease in East Africa and support both clinical practice and the planning of future studies.

## 2. Materials and Methods

### 2.1. Study Design and Setting

A hospital-based, retrospective cross-sectional study was conducted at the Urology Department of Muhimbili National Hospital (MNH), Dar es Salaam, Tanzania. MNH, established in 1910, is the largest tertiary referral and university teaching hospital in Tanzania. Affiliated with the Muhimbili University of Health and Allied Sciences (MUHAS), it is the main referral destination in eastern Africa for complex urological and histopathological management [20,21]. This study adhered to the Strengthening the Reporting of Observational Studies in Epidemiology (STROBE) guidelines for cross-sectional studies [22].

### 2.2. Study Population and Eligibility Criteria

The study population comprised adult male patients who presented to the MNH urology clinics with LUTSs and subsequently underwent prostatic biopsy during the 12-month study period (1 January 2023 to 31 December 2023). Inclusion criteria were as follows: (i) male sex; (ii) age ≥ 50 years; (iii) documented clinical presentation of LUTSs; and (iv) availability of definitive histopathological results from prostatic biopsy or surgical specimen. Patients were excluded if they were aged < 50 years (to minimize confounding from non-age-related urological pathology), had incomplete medical records, lacked histopathological confirmation, or had declined participation.

It should be noted that two patients were retained in the final dataset despite unavailable biopsy results, as their records were otherwise complete and their clinical data were relevant to the overall descriptive analysis; these cases are reported separately in in the histopathological results section and were excluded from the chi-square association analyses. Comorbidity data for all participants, including those without a formally completed questionnaire, were ascertained through systematic retrospective review of clinical consultation notes, discharge summaries, pharmacy prescription records, and nursing assessment documentation within the medical archives, applying a consistent multi-source approach across the entire cohort.

### 2.3. Sample Size Determination

During the study period, a total of 260 patients met the eligibility criteria. The minimum required sample size was calculated using Slovin’s formula for finite populations, *n* = *N*/[1 + *N*(*e*)^2^], where N represents the population size (260) and e denotes the margin of error set at 0.05 to achieve a 95% confidence level. The resulting minimum sample size was 156 patients. Following data cleaning and exclusion of incomplete records, the final analytical cohort comprised 133 patients with complete datasets.

### 2.4. Sampling Strategy and Data Collection

A simple random sampling technique was employed to minimize selection bias. A complete sampling frame was constructed by listing all eligible patients meeting the inclusion criteria. Each patient was assigned a unique identification number, and participants were selected using a computer-generated random number table. Data were systematically retrieved through a retrospective review of patient medical records, surgical theater registers, and histopathology reports. A dedicated digital data extraction instrument was developed using Google Forms to capture all variables consistently and reduce transcription errors.

### 2.5. Study Variables

The primary outcome variable was histopathological diagnosis (benign vs. malignant) from prostatic biopsy. Independent variables included patient demographics (age), comorbid conditions (hypertension, diabetes mellitus, recurrent urinary tract infection [UTI], schistosomiasis), IPSS individual symptom components (incomplete emptying, urinary frequency, intermittency, weak stream, straining, nocturia, urge incontinence, hesitancy, and post-void dribbling), overall IPSS severity classification (moderate: 8–19; severe: 20–35), serum PSA level (ng/mL), and prostate volume (cm^3^).

### 2.6. Statistical Analysis

Collected data were entered into Microsoft Excel for initial cleaning and validation, followed by formal statistical analysis using the Statistical Package for Social Sciences (SPSS) version 26.0 (IBM Corp., Armonk, NY, USA). Continuous variables are reported as mean (SD); categorical variables as frequencies and proportions. Associations between categorical variables were evaluated using the Pearson chi-square test. All statistical tests were two-tailed, and a *p*-value of <0.05 was considered statistically significant.

### 2.7. Ethical Considerations

Ethical approval was obtained from the Ethical Review Board of the University of Medical Sciences and Technology, Khartoum, Sudan (Reference No.: 202301432; date of approval: 27 February 2024). Given the retrospective nature of data collection from anonymized medical archives, the requirement for individual informed consent was waived. Official permission to access patient records was granted by MNH management. Patient confidentiality was maintained throughout. The investigation was conducted in accordance with the principles of the Declaration of Helsinki [23].

## 3. Results

### 3.1. Demographic Characteristics

A total of 133 patients with complete records were included in the final analysis. The largest proportion of patients (39.8%) was in the 70 to 79-year age bracket, followed by the 60–69-year group (34.6%), the 50–59-year group (18.0%), and patients aged 80 years and above (7.5%). The demographic profile of the study participants is presented in Table 1.

### 3.2. Comorbid Conditions

The majority of patients (41.14%) reported no chronic disease. Among those with documented comorbidities, hypertension was the most prevalent (31.65%), followed by diabetes mellitus (12.03%). Recurrent urinary tract infections and schistosomiasis were each reported in 7.59% of the cohort (Table 2).

### 3.3. IPSS Symptom Profile

Among the individual IPSS components, urinary frequency and weak stream were the most prevalent symptoms, each reported by 78.2% of patients. Incomplete emptying was present in 64.7%, nocturia in 62.4%, straining in 54.9%, urge incontinence in 45.1%, and both intermittency and post-void dribbling in 44.4% of patients. Hesitancy was the least commonly reported symptom (38.3%). The detailed IPSS symptom profile is summarized in Table 3.

### 3.4. Serum PSA Levels and Prostate Volume

The mean serum PSA level was 465.12 ng/mL (SD = 1610.11), with values ranging from 0.24 to 10,000 ng/mL, reflecting the wide clinical spectrum of the study population. Nearly half of the patients (43.6%) had PSA values within the 0–10 ng/mL range, while 37.6% had levels exceeding 40 ng/mL (Table 4 and Table 5). The mean prostate volume was 80.62 cm^3^ (SD = 75.61), and three-quarters of patients (75.9%) had prostate volumes above 40 cm^3^ (Table 6). The wide dispersion in both PSA values and prostate volumes confirms that this cohort encompassed a broad spectrum of disease severity, from early symptomatic presentation to advanced pathology, which is consistent with the referral-level case mix expected at a national tertiary center.

### 3.5. Histopathological Biopsy Outcomes

Histopathological analysis of prostatic biopsy specimens revealed that 57.9% (n = 77) of patients had benign findings consistent with BPH, while 40.6% (n = 54) were diagnosed with prostate cancer. Two patients (1.5%) did not have biopsy results available (Table 7).

### 3.6. IPSS Severity Classification

Classification of patients by IPSS severity demonstrated that 59.4% exhibited severe symptoms (IPSS 20–35), while 40.6% had moderate symptoms (IPSS 8–19). No patients in this cohort had mild symptoms, consistent with the referral nature of the study population (Table 8).

### 3.7. Association Between Individual IPSS Symptoms and Biopsy Outcomes

Chi-square analysis showed statistically significant associations between several IPSS symptoms and biopsy outcomes (Table 9). Incomplete emptying (*p* = 0.011), urinary frequency (*p* = 0.014), weak stream (*p* = 0.022), nocturia (*p* = 0.001), urge incontinence (*p* = 0.004), and post-void dribbling (*p* < 0.001) all demonstrated significant associations with biopsy diagnosis. In contrast, intermittency (*p* = 0.574), straining (*p* = 0.056), and hesitancy (*p* = 0.181) did not reach statistical significance. The most striking contrast was seen for post-void dribbling, which was present in 58.4% of benign cases but only 25.9% of malignant cases, and for nocturia, present in 72.2% of benign versus 44.4% of malignant patients, differences that have direct implications for clinical triage. Particularly, among patients with benign histopathology, the presence of these symptoms was consistently higher than among those with malignant diagnoses, suggesting that obstructive and irritative LUTSs are more strongly associated with BPH than with malignancy in this cohort.

### 3.8. Association Between IPSS Severity and Biopsy Outcomes

A statistically significant association was observed between IPSS severity classification and biopsy outcomes (*p* < 0.001) (Table 10). Among patients with malignant biopsies, 63.0% (34/54) had moderate IPSSs, whereas 73.4% (58/79) of those with benign histopathology had severe IPSS scores. This inverse pattern suggests that patients with prostate cancer in this cohort tended to present with comparatively less severe LUTSs than those with BPH.

## 4. Discussion

This study examined the relative prevalence of histologically confirmed BPH and prostate cancer among men presenting with LUTSs at Tanzania’s principal tertiary referral center. The distribution of histopathological diagnoses, with 57.9% of patients found to have benign prostatic pathology and 40.6% diagnosed with prostate cancer, confirms the predominance of BPH as the principal cause of LUTSs in this referral population, while simultaneously revealing a notably high burden of malignant disease. This dual burden matters most in settings where a comprehensive urological workup cannot be guaranteed.

The demographic profile of our study cohort, with the majority of patients (39.8%) falling within the 70–79-year age group, fits the established age profile of both conditions, which rarely present symptomatically before the sixth decade [4,6]. This age distribution aligns with findings from other East African studies, including those from the same institution [11,20], and reflects the typical age at presentation for symptomatic prostatic disease in sub-Saharan Africa. The fact that 74.4% of patients were aged 60 years or older further reinforces the critical importance of targeted urological screening and evaluation in this demographic.

The comorbidity profile of our cohort merits discussion. While the majority of patients (41.14%) had no documented chronic disease, hypertension (31.65%) and diabetes mellitus (12.03%) were the most prevalent comorbidities. This is worth noting, as there is growing evidence linking metabolic disorders to prostatic disease. Several recent studies have identified metabolic syndrome, hypertension, and type 2 diabetes as potential risk factors for BPH development and progression [7,24]. Liu et al. (2024) demonstrated significant associations between multimorbidity and LUTSs secondary to BPH in a large Chinese cohort [25]. In our study, however, no statistically significant correlation was found between individual comorbidities and IPSS symptom profiles, a finding possibly explained by our modest sample size or the inherent limits of retrospective comorbidity recording.

The remarkably high mean serum PSA of 465.12 ng/mL observed in this study requires cautious interpretation. This markedly elevated mean is likely driven by extreme outliers, patients with advanced, metastatic prostate cancer presenting with PSA values up to 10,000 ng/mL. The wide standard deviation (1610.11) and the observation that nearly half (43.6%) of patients had PSA levels within the normal-to-mildly elevated range (0–10 ng/mL) confirm the rightward skew in the distribution. Similar patterns of extremely elevated PSA at presentation have been documented in other sub-Saharan African studies [9,10,11], reflecting the characteristically late-stage presentation of prostate cancer in the region. These data make a case for earlier, more systematic PSA-based screening in Tanzania and comparable settings [12,15,26,27].

The mean prostate volume of 80.62 cm^3^, with three-quarters of patients exceeding 40 cm^3^, suggests most men in this cohort had substantially enlarged prostates. This is consistent with the referral nature of MNH, where patients presenting for urological evaluation and biopsy typically have more advanced disease compared with primary care populations. The predominance of enlarged prostates also correlates with the high prevalence of severe IPSSs (59.4%), as prostate volume is a recognized determinant of LUTS severity [4,14].

Among the individual IPSS symptom components, urinary frequency and weak stream were the most prevalent, each affecting 78.2% of the cohort. These are the classic irritative and obstructive features of LUTSs, and their high frequency mirrors what other studies have reported in similar prostatic enlargement populations [1,2,14]. Lori et al. (2025), in a study conducted at the same institution, similarly reported a high prevalence of obstructive and irritative symptoms among men with BPH [20].

One of the more clinically relevant findings was the difference in symptom profiles between the benign and malignant groups. Several individual symptoms, nocturia (*p* = 0.001), urge incontinence (*p* = 0.004), and post-void dribbling (*p* < 0.001), demonstrated statistically significant associations with biopsy diagnosis. Notably, these symptoms were more prevalent among patients with benign histopathology, suggesting that severe obstructive and irritative symptoms may be more characteristic of BPH than of localized prostate cancer in this population. This makes mechanistic sense: BPH produces LUTSs chiefly through bladder outlet obstruction of the prostatic urethra, whereas early-to-moderate prostate cancer may not produce significant urinary obstruction until locally advanced [4,13].

The inverse relationship between IPSS severity and malignant histopathology, whereby 63% of patients with prostate cancer had moderate symptom scores, while the majority of BPH patients had severe scores, has real clinical relevance. This finding is consistent with Chandra Engel et al. (2020), who reported that LUTSs were not independently associated with increased prostate cancer risk in men aged 50–69 years with elevated PSA [28]. Conversely, Martin et al. (2008) found that severe LUTSs were associated with increased prostate cancer risk in the HUNT 2 cohort, though this association may have been confounded by detection bias [29]. This apparent paradox may be explained by the tendency of aggressive, high-grade tumors to declare themselves through rising PSA or systemic symptoms rather than the slow obstructive voiding pattern typical of BPH.

Beyond the prostate itself, there is growing interest in the relationship between LUTSs and systemic inflammatory conditions. Recent evidence has documented a significant epidemiological association between lower urinary tract symptoms and inflammatory bowel disease (IBD), with shared immune-mediated pathways proposed as a potential underlying mechanism [30]. Advances in minimally invasive surgical approaches for LUTSs also offer potential relevance in this context, given that symptom relief strategies may benefit patients with overlapping urological and inflammatory conditions [31]. While these systemic associations were beyond the scope of the present study, clinicians evaluating men with LUTSs, particularly in settings where multimorbidity is prevalent, should be aware that urinary symptoms do not always reflect isolated prostatic disease.

Several limitations of this study deserve acknowledgment. First, the retrospective nature of data collection introduces the possibility of information bias and precludes definitive causal inferences. Reliance on medical records may result in under-ascertainment of comorbidities and incomplete documentation of symptom severity. In particular, IPSS data were derived from clinical records rather than prospectively administered questionnaires, meaning that symptom documentation varied between clinicians and may not have captured the full severity or duration of individual symptoms with consistent precision. Second, the sample size of 133 patients, while adequate for descriptive purposes, limits the statistical power to detect modest associations and restricts the precision of subgroup analyses. Third, as a single-center study conducted at a tertiary referral hospital, the findings may not be generalizable to primary care settings or community-dwelling populations, where the prevalence and severity of prostatic disease may differ considerably [16]. Fourth, the absence of mild IPSS cases in this cohort reflects referral bias and limits the applicability of these findings to the full spectrum of LUTS severity. Finally, certain clinically important variables, including Gleason scores, staging information, and treatment outcomes, were not systematically analyzed, each an avenue for future work.

Despite these limitations, this study adds to what remains a thin evidence base on prostatic disease in East Africa. The findings highlight the high burden of both BPH and prostate cancer among men presenting with LUTSs at tertiary facilities in Tanzania and underscore the importance of comprehensive histopathological evaluation in all symptomatic patients. Future prospective multicenter studies with larger sample sizes and longitudinal follow-up are warranted to elucidate the temporal relationships between LUTSs, comorbid conditions, and prostatic pathology in this population. Additionally, studies investigating the cost-effectiveness of integrated BPH and prostate cancer screening programs in resource-limited settings would carry real public health relevance [16,18].

## 5. Conclusions

This study demonstrates that BPH is the predominant histopathological diagnosis among men presenting with LUTSs at Muhimbili National Hospital, accounting for 57.9% of cases, while prostate cancer represents a substantial minority at 40.6%. Certain individual LUTSs, most notably nocturia, urge incontinence, and post-void dribbling, exhibited statistically significant associations with biopsy outcomes, and an inverse relationship between IPSS severity and malignant histopathology was observed. These results reinforce the need for routine tissue diagnosis in all men presenting with LUTSs, particularly in sub-Saharan African settings where late-stage prostate cancer presentation remains a significant challenge. Men with moderate LUTSs and an elevated PSA should not be reassured by the absence of severe symptoms; occult malignancy remains a real possibility and biopsy should be considered.

## Figures and Tables

**Table 1:** Age distribution of study participants (N = 133).

Age Group (Years)	Frequency (n)	Percentage (%)
50–59	24	18.0
60–69	46	34.6
70–79	53	39.8
≥80	10	7.5
Total	133	100.0

**Table 2:** Distribution of comorbid conditions among study participants.

Comorbid Condition	Frequency (n)	Percentage (%)
No chronic disease	55	41.14
Hypertension	42	31.65
Diabetes mellitus	16	12.03
Recurrent UTI	10	7.59
Schistosomiasis	10	7.59

**Table 3:** Distribution of individual IPSS symptoms among study participants (N = 133).

IPSS Symptom	Absent, n (%)	Present, n (%)
Incomplete emptying	47 (35.3)	86 (64.7)
Urinary frequency	29 (21.8)	104 (78.2)
Intermittency	74 (55.6)	59 (44.4)
Weak stream	29 (21.8)	104 (78.2)
Straining	60 (45.1)	73 (54.9)
Nocturia	50 (37.6)	83 (62.4)
Urge incontinence	73 (54.9)	60 (45.1)
Hesitancy	82 (61.7)	51 (38.3)
Post-void dribbling	74 (55.6)	59 (44.4)

**Table 4:** Descriptive statistics for serum PSA and prostate volume (N = 133).

Variable	Minimum	Maximum	Mean	SD
PSA (ng/mL)	0.24	10,000.00	465.12	1610.11
Prostate volume (cm^3^)	4.00	609.00	80.62	75.61

**Table 5:** Distribution of serum PSA levels (N = 133).

PSA Range (ng/mL)	Frequency (n)	Percentage (%)
0–10	58	43.6
10–20	12	9.0
20–30	8	6.0
30–40	5	3.8
>40	50	37.6
Total	133	100.0

**Table 6:** Distribution of prostate volume (N = 133).

Prostate Volume (cm^3^)	Frequency (n)	Percentage (%)
0–10	1	0.8
10–20	7	5.3
20–30	14	10.5
30–40	10	7.5
>40	101	75.9
Total	133	100.0

**Table 7:** Distribution of histopathological biopsy outcomes (N = 133).

Histopathological Diagnosis	Frequency (n)	Percentage (%)
Benign (BPH)	77	57.9
Malignant (PCa)	54	40.6
Biopsy result unavailable	2	1.5
Total	133	100.0

**Table 8:** Distribution of IPSS severity classification (N = 133).

IPSS Severity	Frequency (n)	Percentage (%)
Moderate (8–19)	54	40.6
Severe (20–35)	79	59.4
Total	133	100.0

**Table 9:** Cross-tabulation between individual IPSS symptoms and histopathological biopsy outcomes (N = 133).

IPSS Symptom	Response	Benign	Malignant	No Biopsy	*p*-Value
Incomplete emptying	No	19	27	1	***0.011***
	Yes	58	27	1	
Urinary frequency	No	10	18	1	***0.014***
	Yes	67	36	1	
Intermittency	No	40	33	1	*0.574*
	Yes	37	21	1	
Weak stream	No	11	18	0	***0.022***
	Yes	66	36	2	
Straining	No	29	29	2	*0.056*
	Yes	48	25	0	
Nocturia	No	20	30	0	***0.001***
	Yes	57	24	2	
Urge incontinence	No	33	39	1	***0.004***
	Yes	44	15	1	
Hesitancy	No	43	37	2	*0.181*
	Yes	34	17	0	
Post-void dribbling	No	32	40	2	***<0.001***
	Yes	45	14	0	

Bold italic *p*-values denote statistical significance (*p* < 0.05). Italic alone denotes non-significant *p*-values.

**Table 10:** Cross-tabulation between IPSS severity and histopathological biopsy outcomes (N = 133).

Biopsy Outcome	Moderate IPSS, n	Severe IPSS, n	*p*-Value
Benign	19	58	***<0.001***
Malignant	34	20	
Biopsy result unavailable	1	1	

Bold italic *p*-values denote statistical significance (*p* < 0.05).

## Data Availability

The data supporting the findings of this study are available from the corresponding author upon reasonable request.
